# Evidence-based Pharmacological Treatment in the Maintenance Phase of the Type I Bipolar Disorder: Anticonvulsants or Antipsychotics?

**DOI:** 10.1192/j.eurpsy.2022.1029

**Published:** 2022-09-01

**Authors:** O. Vasiliu

**Affiliations:** Dr. Carol Davila University Emergency Central Military Hospital, Psychiatry, Bucharest, Romania

**Keywords:** maintenance phase, moodstabilizers, bipolar disorder

## Abstract

**Introduction:**

Type I bipolar disorder (BDI) is characterized by a chronic evolution, with recurrent mood episodes that severely disrupt the overall functionality and quality of patients’ life. An adequate maintenance treatment is necessary to prevent relapses and to improve the functional prognosis of these patients.

**Objectives:**

To find data regarding the most evidence-based therapeutic strategies in the maintenance phase of BDI.

**Methods:**

A literature review was performed through the main electronic databases (PubMed, CINAHL, SCOPUS, EMBASE) using the search paradigm “type I bipolar disorder” AND “mood stabilizers” AND “antipsychotics” AND “anticonvulsants”. All papers published between January 2000 and August 2021 were included.

**Results:**

The main recommendation is to continue in the maintenance phase the same medication that has proven its efficacy and tolerability in the acute phase. In BDI the most evidence-supported pharmacological approaches for the maintenance phase were lithium, valproate, lamotrigine, and carbamazepine as anticonvulsants/mood stabilizers, as well as olanzapine, quetiapine, and aripiprazole as antipsychotics. Lithium and valproate have been associated with positive influence over neuroplasticity, while antipsychotics have considerably higher metabolic adverse events. Monotherapy is recommended, but drugs associations are frequently met in clinical practice. There are no consistent data about the superiority of one class over the other, but lithium has a proven effect of decreasing the suicide rate in this population.

**Conclusions:**

Both anticonvulsants and antipsychotics are used in the maintenance phase of the BDI, without significant differences in the efficacy rates. However, benefits and risks should be weighted for each class and each individual agent recommended.

**Disclosure:**

No significant relationships.

